# The Surgical Management of Chronic Thromboembolic Pulmonary Hypertension

**DOI:** 10.3390/jcm14196862

**Published:** 2025-09-28

**Authors:** Kevin C. McGann, Chen Chia Wang, John M. Trahanas, Swaroop Bommareddi, Brian Lima, Awab Ahmad, Clifford W. Chin, Ivan M. Robbins, Meredith E. Pugh, Anna R. Hemnes, Blake Funke, Ashish S. Shah, Aaron M. Williams

**Affiliations:** 1 Department of Cardiac Surgery, Vanderbilt University Medical Center, Nashville, TN 37232, USA; kevin.mcgann@vumc.org (K.C.M.); chen.chia.wang@vanderbilt.edu (C.C.W.); john.trahanas@vumc.org (J.M.T.); swaroop.bommareddi@vumc.org (S.B.); brian.lima@vumc.org (B.L.); awab.ahmad@vumc.org (A.A.); clifford.chin.1@vumc.org (C.W.C.); ashish.s.shah@vumc.org (A.S.S.); 2 Department of Pulmonology, Vanderbilt University Medical Center, Nashville, TN 37232, USA; ivan.robbins@vumc.org (I.M.R.); meredith.e.pugh@vumc.org (M.E.P.); anna.r.hemnes@vumc.org (A.R.H.); blake.e.funke@vumc.org (B.F.)

**Keywords:** chronic thromboembolic pulmonary hypertension (CTEPH), pulmonary thromboendarterectomy (PTE), pulmonary hypertension (PH), surgical technique, pulmonary endarterectomy (PEA), thromboembolic disease

## Abstract

Chronic thromboembolic pulmonary hypertension (CTEPH) is a type of pulmonary hypertension due to unresolved thromboembolic disease that presents with signs of pulmonary artery obstruction and right heart dysfunction. Pulmonary thromboendoarterectomy (PTE) with deep hypothermic circulatory arrest remains the standard of care for the treatment of CTEPH, with significant improvements in symptoms and functional status after surgery. This review outlines the diagnostic workup, considerations during operative planning, surgical technique, and postoperative management of CTEPH patients.

## 1. Introduction

Chronic thromboembolic pulmonary hypertension (CTEPH) is pulmonary hypertension (PH) due to underlying, unresolved thromboembolic disease leading to pulmonary artery obstruction [1]. CTEPH arises as a complication of acute pulmonary embolism (PE) [2], and has an estimated incidence of between 1 and 5% within acute PE survivors [3,4,5,6]. In terms of timing, the development of CTEPH is rare after two years of the acute PE episode [5]. Still, as many as 40–60% of CTEPH patients have no known history of acute venous thromboembolism (VTE) [7,8]. CTEPH falls under Group 4 PH in the World Health Organization’s PH classification schema, which comprises PH due to the thromboembolic occlusion of pulmonary vasculature, distinguishing it from other etiologies of PH such as vascular remodeling of pulmonary arteries (Group 1), left-sided heart dysfunction (Group 2), and intrinsic lung disease or hypoxemia (Group 3) [9]. CTEPH is associated with high morbidity and mortality, but recent advances in both medical and surgical management have improved outcomes [10,11,12]. Without treatment, the median survival of CTEPH is less than 3 years [13,14]. While non-medical management options have expanded and have extended the median survival to over 5 years, surgery remains the standard of care [15,16]. Pulmonary thromboendarterectomy (PTE) is the surgical standard for CTEPH, and has been shown to improve hemodynamic and functional outcomes as well as long term survival [17,18,19]. Outcomes after PTE have also improved over time [20]. This review will focus on the surgical strategies and techniques in PTE for CTEPH, as well as the associated outcomes and potential management of complications.

## 2. Pathophysiology and Genetics

The precise pathophysiology of CTEPH is unclear, but several hypotheses exist. The commonly accepted theory suggests that CTEPH arises from prior PE episodes, but it has also been postulated that CTEPH could arise due to in situ thrombosis or lysis-resistant fibrin variants [21,22]. This alternate hypothesis may explain why close to 40% of patients with CTEPH do not report a history of acute PE [23,24]. Furthermore, there is no correlation between the mechanical obstruction of the chronic thromboembolic disease (CTED) and the severity of the pulmonary vascular resistance (PVR), unlike that with acute PE [25]. Known risk factors for CTEPH are similar to those for acute PE, and include hypercoagulable states, malignancy, and heart failure [6,26,27,28,29,30]. Specifically, elevated levels of factor VIII and the presence of antiphospholipid antibodies have been associated with CTEPH [31,32], whereas deficiencies in antithrombin, protein C, and protein S have not [33]. In addition, a previous history of PE (odds ratio 19.0), younger age (odds ratio 1.79 per decade decrease), a larger perfusion defect (odds ratio 2.22 per decile decrease in perfusion), and idiopathic PE at presentation (odds ratio 5.70) have been identified as risk factors for CTEPH [4]. Additional clinical risk factors include prior splenectomy, ventriculoatrial shunt, infected pacemaker, and inflammatory bowel disease [28]. Experimental studies highlight that endothelial dysfunction, dysfunctional angiogenesis, staphylococcal infection, and other inflammatory and immunological mechanisms have also been associated with CTEPH [34,35,36].

## 3. Clinical Presentation and Diagnosis

As CTEPH is a chronic process, symptoms are usually mild initially and worsen over time, and patients may not present until the later stages of disease. Presenting symptoms are usually nonspecific features of PH, such as progressive dyspnea, exercise intolerance, dizziness, and fatigue [37,38]. Patients may also present with signs of right ventricular dysfunction indicative of later-stage disease, such as peripheral edema, exertional angina, and syncope. A distant history of acute PE or other venous thromboembolic events in addition to suggestive symptoms such as pleuritic chest pain or lower extremity discomfort may also be reported [24]. On physical exam, an accentuated second heart sound, S4 gallop, and systolic ejection click over the pulmonic area may be auscultated [39]. A more unique, CTEPH-specific physical exam finding is the presence of flow murmurs over the lung fields as a result of medium/large pulmonary artery obstruction [40]. These are high-pitched, blowing bruits most commonly observed in the posterior lung fields and frequently occurring only during breath-holding. Importantly, these flow murmurs have not been described in other groups of PH, though they are only present in roughly 30% of patients with CTEPH [40]. Hypoxemia is another common finding in CTEPH due to ventilation–perfusion mismatch [41]. However, most symptoms are not specific to CTEPH, and therefore the diagnosis of CTEPH is often not made until approximately 14 months after symptom onset [27,42].

Delayed diagnosis of CTEPH can result in progressive symptoms and poor utilization of inappropriate treatment and testing resources. During this time interval, progression of CTEPH can lead to worsening small vessel disease, which may ultimately result in worse clinical outcomes. When suspecting CTEPH, dedicated diagnostic testing should be reserved for those with new or persistent symptoms despite at least 3 months of anticoagulation therapy. There are several imaging modalities that may be pursued in the workup of CTEPH. Electrocardiogram is not sensitive enough to rule out CTEPH but can highlight some subtle signs of the disease, including right axis deviation, right ventricular hypertrophy, right bundle branch block, and QTc prolongation [43]. Chest radiographs may be normal in early CTEPH but can often show characteristic features of PH such as cardiomegaly, pulmonary artery enlargement, chronic volume loss, and atelectasis or effusion in later-stage CTEPH [7,8]. V/Q scanning has a high sensitivity (96–97%) and specificity (90–95%) for CTEPH, and is therefore the first-line screening tool to diagnose CTEPH [44,45,46]. Therefore, CTEPH can accurately be excluded in the setting of a normal V/Q scan. However, V/Q scanning may underestimate the degree of central pulmonary vascular obstruction, and may not distinguish CTEPH from other disorders that can cause large perfusion defects such as pulmonary artery sarcoma or extrinsic pulmonary vascular compression [47].

**Figure 1:**
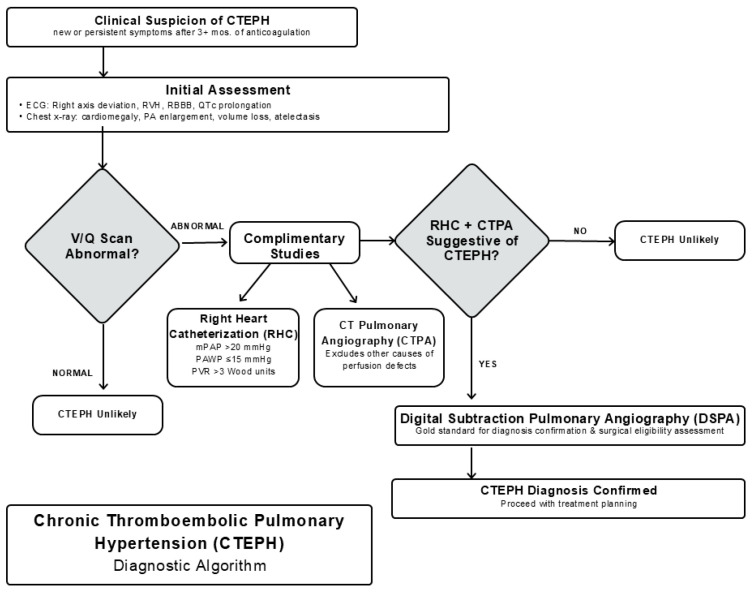
Diagnostic algorithm for the evaluation of CTEPH. RVH = right ventricular hypertrophy; RBBB = right bundle branch block; mPAP = mean pulmonary artery pressure; PAWP = pulmonary artery wedge pressure; PVR = pulmonary vascular resistance.

Importantly, right heart catheterization (RHC) coupled with CT pulmonary arteriography (CTPA) is often required as a complementary study to V/Q scanning [48,49]. RHC is necessary to confirm the diagnosis and provide hemodynamic parameters that aid in predicting subsequent prognosis [50]. On RHC, the classic diagnostic criteria for precapillary PH is specified by mean pulmonary artery pressure (mPAP) > 20 mmHg, pulmonary artery wedge pressure ≤ 15 mmHg, and pulmonary vascular resistance (PVR) of ≥3 Woods units, as defined the European Respiratory Society [9]. CTPA is also a crucial study to complement RHC and V/Q scanning [48,49]. CTPA helps exclude other possible causes of perfusion defects seen on V/Q scanning and confirms the distribution of thromboembolic disease to guide surgical planning [51]. In patients with both V/Q scanning and CTPA suggestive of CTEPH, digital subtraction pulmonary angiography (DSPA) is the gold standard in confirming the diagnosis and determining surgical eligibility [39,52]. A basic overview of a potential diagnostic algorithm for the workup of CTEPH is provided in Figure 1.

There are several other less commonly ordered tests that may be useful in the workup of CTEPH. Cardiopulmonary exercise testing (CPET) is an important, noninvasive method of identifying patients with milder symptoms of CTEPH for further evaluation. Findings of CTEPH in CPET include decreased peak oxygen uptake (VO_2_), increased VE/VCO_2_ (minute ventilation/CO_2_ production per minute) ratio, greater alveolar–arterial oxygen gradient, and reduced end-tidal CO_2_ at anaerobic threshold [48,53,54,55,56]. These CPET findings indicate inefficient ventilation and impaired pulmonary perfusion, and can aid in monitoring and prediction of CTEPH severity [55]. Lastly, magnetic resonance imaging (MRI) is a new, emerging tool for the diagnosis and monitoring of CTEPH patients. MRI provides high-resolution assessments of right ventricular morphology and function, pulmonary hemodynamics, and pulmonary perfusion defects [50,57,58]. However, recent comparisons demonstrate that CTPA is still superior to MRI at detecting occlusions in the pulmonary vasculature, especially beyond the segmental level [59,60]. Despite this, MRI has grown to be a valuable tool in monitoring of right ventricular function and pulmonary hemodynamics following the surgical management of CTEPH [50,58]. Lastly, the noninvasive nature and lack of radiation exposure in MRI are both important benefits of this modality over other options [61].

## 4. Multidisciplinary Chronic Thromboembolic Pulmonary Hypertension Teams

Given the historically poor outcomes in the treatment of acute PE that may lead to eventual CTEPH, there has been increasing adoption of multidisciplinary teams to help establish consensus for CTEPH treatment. These teams typically consist of pulmonologists, pulmonary hypertension specialists, interventionalists, and cardiothoracic surgeons who collectively decide the best treatment approaches, whether medical, catheter-based, or surgical. In the acute setting, while catheter-directed or surgical thrombectomy may decrease long-term CTEPH risk by limiting residual thrombi, their primary objective is the improvement of prognosis at the time of presentation [62]. Mechanical support options are also typically discussed. With the adoption of these teams, some early data suggests improvement in patient outcomes [63,64]. It should be noted that balloon pulmonary angioplasty (BPA) has demonstrated safety and efficacy for inoperable CTEPH in multicenter randomized trials, but falls outside of the scope of this review and will not be discussed in detail here [65,66].

## 5. Basics of Medical Management

The initial step in CTEPH management is the immediate initiation of anticoagulation therapy, assuming the patient is not at high risk of bleeding [67]. Initial therapy is usually intravenous unfractionated heparin, subcutaneous low molecular weight heparin, or a direct oral anticoagulant (DOAC). Long-term anticoagulation is achieved either using a vitamin K antagonist (warfarin) or a DOAC, with similar efficacy reported between the two options [68,69,70]. DOACs have been associated with decreased rates of recurrent embolism, even in high risk patients such as oncology patients following acute PE [71]. Anticoagulation should be continued indefinitely, regardless of other interventions that the patient may receive for CTEPH management [67].

For nonsurgical candidates, several adjunct options for medical management have been shown to improve hemodynamics and functional outcomes when used in combination with anticoagulation. Various trials have shown that inhaled or subcutaneous prostacyclin analogues improve 6 min walk distances and quality of life (AIR and CTREPH trials, respectively) [72,73]. The BENEFiT trial demonstrated that bosentan therapy, an endothelial receptor antagonist, led to significant improvements in pulmonary vascular resistance, cardiac index, and brain-type natriuretic peptide levels [74]. Recently, the CHEST-1 and CHEST-2 trials demonstrated that riociguat, a guanylate cyclase stimulator, significantly improved exercise capacity and pulmonary vascular resistance in patients with inoperable CTEPH [75]. Riociguat is currently the only drug approved by the United States Food and Drug Administration for CTEPH, and along with other pulmonary vasodilator therapies, is considered a viable treatment option for disease that is inoperable or refractory to surgery [76,77].

## 6. Considerations in Patient Selection for Surgery

Like most other surgical therapeutic options, individual patient factors, medical comorbidities, and the distribution and severity of the given disease presentation determine suitability for endarterectomy in CTEPH. As stated previously, a multidisciplinary evaluation incorporating the expertise of cardiologists, pulmonologists, radiologists, and cardiothoracic surgeons is essential. The degree of PH, right ventricular dysfunction, and obstruction should be evaluated critically, and in general, all symptomatic patients with corresponding obstruction should at least be considered for surgical treatment despite the degree of corresponding PH and right heart dysfunction [78].

To this end, the indications for surgical intervention have been debated extensively and largely remain without strict boundaries. Patients with a defined history of thromboembolic disease, no evidence of right heart failure, minimal comorbidities, ample functional status, clear and bilateral disease on imaging, and lower PVR in proportion to the observed distribution and degree of obstruction are generally deemed lower-risk operative candidates (Table 1) [37]. Disease located in the larger, more central and surgically accessible pulmonary artery branches (e.g., main, lobar, proximal segmental) is also more readily suited for PTE compared to disease predominantly confined to the distal vasculature [78]. Nonetheless, even high-risk patients with more challenging distributions of segmental and subsegmental obstruction may still be operative candidates at select high-volume centers. In general, centers are considered to have surgical expertise if they perform at least 50 cases annually and have an in-hospital mortality of less than 5% [79].

The burden of disease in CTEPH is typically categorized based on anatomic location. Level 1 involves disease starting in the main PA, level II involves disease starting in the lobar PA, level III involves disease starting in the segmental PA, and level IV disease originates in the subsegmental PA branches. While levels I and II are typically accessed with more surgical ease, disease in levels III and IV remains much more difficult to access and should only be operated on at select high-volume centers [80]. Level IV disease is considered by some to be surgically inaccessible, although in highly select centers, surgical intervention may be feasible [80]. Patients with disease localized in the segmental and subsegmental pulmonary arteries on imaging are candidates for PTE, with both excellent early and late outcomes [81]. Although the correlation between disease burden on preoperative imaging and the severity of PVR remains encouraging, this is not a reliable parameter, as imaging underestimates the amount of disease [82]. Furthermore, patients can have a dramatic improvement in hemodynamics despite only a limited amount of disease removed [82]. Up to 90% of patients have been shown to be surgical candidates despite being high-risk with medical comorbidities and right heart failure [83].

**Figure 2:**
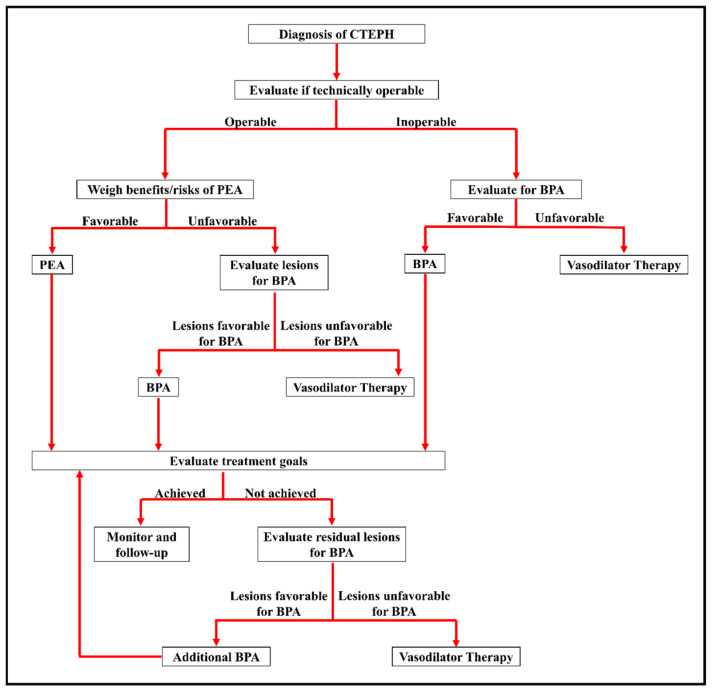
Treatment evaluation algorithm for CTEPH. BPA = balloon pulmonary angioplasty; PEA = pulmonary endarterectomy. Adapted from Ref. [86]. 2019, American College of Cardiology Foundation.

It is our practice to pursue PTE as first-line therapy for CTEPH patients with technically operable disease who are appropriate surgical candidates, in accordance with AHA consensus guidelines [84]. That being said, it is worth noting that the role of BPA in CTEPH management continues to evolve and we anticipate it will continue to enrich the multidisciplinary coordination between surgical and interventional teams. Currently, we explore BPA as a valuable treatment modality in surgically inaccessible disease and/or residual PH following surgery. In such patients, BPA appears to offer significant improvements in outcomes when compared to medical therapy alone and shows promise for a growing role in standard treatment approaches [84,85]. An overarching algorithm for treatment option considerations after a diagnosis of CTEPH is established is shown in Figure 2.

## 7. Surgical Technique

PTE with deep hypothermic circulatory arrest remains the standard of care for the treatment of CTEPH.

The operative approach to PTE has been described previously [78,87,88], and here, it will be outlined with particular emphasis on the most critical steps and potential pitfalls. We simplify the overall approach to PTE into the following major technical steps, each of which may vary depending on the specific case and institution:

1. Median sternotomy and pericardiotomy;
2. Establishment of cardiopulmonary bypass;
3. Systemic cooling to 18–20 °C;
4. Right pulmonary thromboendarterectomy with short-interval (<20 min) circulatory arrest;
5. Left pulmonary thromboendarterectomy with short-interval (<20 min) circulatory arrest;
6. Rewarming and closure of patent foramen ovale (if present).

Following coordinated induction of anesthesia, a PA catheter is placed to confirm elevated PA pressures, monitored continuously throughout the operation. This may or may not be able to be placed based on the disease burden. Transesophageal echocardiography (TEE) should also be performed by cardiac anesthesiology at this time to evaluate for a PFO and assess overall right and left heart structure and function.

The surgery begins with a median sternotomy, which enables optimal exposure for central cardiopulmonary bypass (CPB) as well as right and left pulmonary endarterectomies. After longitudinal pericardiotomy and initial dissection are completed, the patient is systemically heparinized. Cannulation for bypass is then performed, typically with bicaval drainage of the superior vena cava (SVC) and inferior vena cava (IVC) and high ascending aortic cannulation. Alternatively, the right atrium may be cannulated directly along with the SVC for venous drainage. Full CPB is instituted and allows for the heart to be emptied. A left ventricular vent is inserted via the right superior pulmonary vein, and a PA vent is inserted into the main PA.

The patient is then systemically cooled to 18–20 °C using the combination of the pump oxygenator and surface cooling measures. The right and left pulmonary arteries are dissected free, and the right pulmonary artery (RPA) is carefully mobilized off the aorta and superior vena cava. After cooling has been completed for typically 1 h, an aortic cross clamp may be applied and antegrade cold blood cardioplegia administered for myocardial protection [78]. This, however, is not common and often not needed. We then use a Henley retractor or a similar type of retractor to open the dissected space between the aorta and SVC for optimal exposure of the RPA.

Longitudinal right pulmonary arteriotomy is performed by the surgeon from the patient’s left side and extended distally past the truncus arteriosus. Any loose thrombus is cleared. The endarterectomy plane is then created between the intimal and medial layers of the vessel wall using a 15-blade scalpel or Beaver blade. With the combination of meticulous traction and suction dissection, this plane is carried circumferentially and followed distally. After confirming systemic cooling temperatures and a reading of zero on bispectral index (BIS) monitoring, hypothermic circulatory arrest is performed in short interval (under 20 min) to facilitate full endarterectomy on the right side. The vessel is everted and each subsegmental branch is carefully followed and freed individually until the specimen “tails off” and becomes free on its own [78]. Periods of 20 min of deep hypothermic circulatory arrest followed by intervals of 10 min of reperfusion are well tolerated [83]. After the specimen is removed, CPB is then reinstated and the arteriotomy is closed with a 6-0 polypropylene suture in a running fashion.

**Figure 3:**
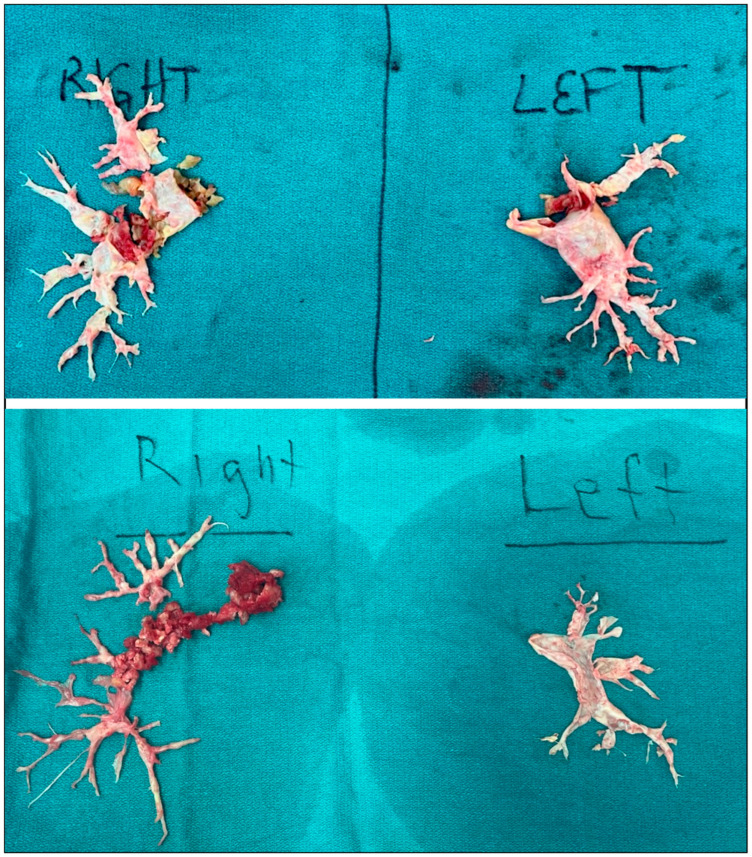
Pulmonary thromboendarterectomy specimens involving level 1 disease from the main pulmonary arteries to the subsegmental pulmonary artery branches.

From the patient’s right side, the surgeon then performs the left pulmonary arteriotomy. This is again performed longitudinally, and here is extended to the pericardial reflection without violation of the pleura. Overly lateral dissection increases the risk of injury to the left phrenic nerve [78]. Again, any thrombus is removed from the vessel, and the appropriate endarterectomy plane is carefully fashioned using a scalpel (Figure 3). Once distal, hypothermic circulatory arrest is again performed in short interval (under 20 min) followed by 10 min intervals of reperfusion for completion of the left-sided endarterectomy. CPB is again reinstated and the left-sided arteriotomy is similarly closed in a running fashion.

After five minutes of reperfusion, the patient is fully rewarmed systemically. During this time, any other indicated cardiac procedures such as closure of a patent foramen ovale can be conveniently performed. If this is this case, the cava can be snared to help minimize blood in the visualized field. It is worth noting that while secondary tricuspid valve dysfunction is commonly observed in CTEPH patients, we do not generally perform tricuspid valve repairs in the absence of anatomic abnormalities. It has been shown that annular dilation secondary to RV dysfunction improves after PTE in CTEPH as RV remodeling leads to the return of valvular competence [89,90]. Ionotropic support and pulmonary vasodilators may be required to support the right heart following PTE. Aggressive diuresis and monitoring for any reperfusion injury and airway bleeding is important after weaning from CPB.

## 8. Surgical Outcomes and Complications

In the short term, the hemodynamic benefits of PTE in CTEPH are often striking and have been well described, with dramatic improvements in measures including PA pressure, PVR, 6 min walk testing, and NYHA functional class [19]. At experienced centers, the in-hospital mortality rate has declined over time and remains under 5% highlighting the safety and validity of PTE as a fundamental treatment modality [20,88,91,92]. In one retrospective study of long-term outcomes in 499 patients undergoing PTE for CTEPH over a 20-year period, overall survival at 5, 10, and 15 years postoperatively was 84.8 ± 1.9%, 77.1 ± 2.7%, and 59.2 ± 5.3%, respectively [93]. Importantly, this study showed that there was a significant and step-wise decline in all-cause mortality at 30 days depending on the period of surgery suggesting that experience in the surgical management of these patients is critically important. For example, all-cause mortality at 30 days was 14.0% among the first two hundred cases performed but then dropped significantly to 7.0% among the next hundred cases and ultimately was as low as 1% in the most recent 99 cases performed. The most common causes of death in these patients included sepsis, multiorgan failure, and low cardiac output syndrome [93]. A more focused analysis of this specific relationship between surgical volume and early mortality for PTE in CTEPH was recently published and similarly highlighted the progressive inverse relationship between the two. In centers performing more than 100 pulmonary endarterectomy procedures annually, early mortality rates were 2.9%, compared to 5.4% in centers with an annual case volume of 51–100 and 6.7% in centers with an annual case volume of 16–50 [94].

Some of the most comprehensive data available on PTE outcomes in CTEPH patients comes from large international registries. A global registry of patients from 34 centers in 20 countries examined the impact of PTE and nonsurgical treatments on long-term survival in CTEPH patients followed from the time of diagnosis between February 2015 and September 2016 until September 2019 [95]. Of the 1009 patients diagnosed with CTEPH during the study window, 605 underwent surgery, and 81% of these patients had a history of VTE. Overall, the three-year survival for PTE was 94%, a 5% increase relative to the earlier European CTEPH registry in a Kaplan–Meier analysis. The reported 30-day mortality rate of 3.2%, which represented a decrease from 4.7% in the earlier study. Consistent with previous outcomes data, PTE was shown to significantly improve hemodynamics among CTEPH patients. PVR decreased 57% and median mean pulmonary arterial pressure dropped from 45 mm Hg to 24 mm Hg following surgery. Notably, the use of pulmonary hypertension drugs, either before or after surgery, was not found to have any significant effect on survival in the PTE group. This global registry analysis provides further data to corroborate the role for PTE in the treatment of patients with operable CTEPH, and points to improved techniques and increased experience in participating centers that contributed to an increased 3-year survival rate relative to the earlier European registry data.

Despite significant improvements in hemodynamic parameters, patients are still at risk for persistent PH following PTE, with prevalence as high as 45% in a single-center European study [96]. This prospective study was conducted from October 2014 until July 2016. Of the 42 identified patients, 31 had complete follow-up hemodynamic studies; 14 (45%) had hemodynamic evidence of persistent PH; 28 patients underwent postoperative lung perfusion MRI; 13 (46%) had persistent PH. However, the degree of lung hypoperfusion was similar in all patients (20% abnormal perfusion in persistent PH, 19% without). The authors concluded that persistent postoperative PH may be predominantly due to microvascular disease. This pathology may favor medical management for distal disease with riociguat, rather than further procedural intervention such as balloon pulmonary angioplasty [75]. Beyond debate surrounding the definition of residual PH after PTE, the more clinically relevant discussion surrounds the appropriate risk stratification of the significant proportion of patients with residual PH post PTE and determination of who would benefit most from additional treatment.

Data from the European groups highlight that a mean pulmonary artery pressure > 38 mmHg and PVR > 425 dynes.sec.cm/5.3 Woods units was associated with increased mortality secondary to right heart failure [97]. As such, this highlights that residual or recurrent PH must continue to be re-evaluated in patients after PTE and to follow them long-term. At present, patients should undergo repeat exercise testing (6 min walk test with CPET), RHC, and imaging within the first year, then at least yearly thereafter.

There are also several other points worth highlighting regarding surgical candidacy. First, despite prior misconceptions, deep hypothermic circulatory arrest does not impact the cognitive function in CTEPH patients post-PTE. Several studies have shown that intermittent deep hypothermic circulatory arrest, which is often required to attain complete removal of the chronic thromboembolic material in the distal PA branches such as level IV, is considered safe [91]. Furthermore, CTEPH patients who underwent PTE had improved cognitive function at 3 and 12 months compared to prior to surgery [91]. This is likely secondary to increased brain perfusion because of improved cardiac output after PTE. Second, there does not appear to be a PVR above which PTE is contraindicated. Although patients with higher PVRs (>12.5 Wood units) appear to be the highest risk, PTE is not contraindicated and these patients may gain the most benefit in the setting of right heart dysfunction [20]. Lastly, older patients above 70 years old also appear to gain as much benefit from PTE compared to those less than 70 years as long as there are no prohibitive comorbidities for surgery otherwise [98].

## 9. The Role of ECMO After PTE

Several of the most severe but rare complications following PTE include reperfusion pulmonary edema, airway hemorrhage, or inability to wean from cardiopulmonary bypass that necessitates extracorporeal membrane oxygenation (ECMO) support. In larger patient cohorts, ECMO may be required following PTE in up to 5% of cases [99,100]. Despite this, the majority of patients are decannulated from ECMO and are eventually discharged, nearing around 70–80% of patients [99,100].

Both types of ECMO, including veno-arterial ECMO (VA-ECMO) and veno-venous ECMO (VV-ECMO), may be required depending on the primary clinical scenario. Before surgery, patients with CTEPH may require VA-ECMO to help support the decompensated right heart prior to PTE or to help facilitate transfer to another center. After PTE, VA-ECMO may also be required to help wean from cardiopulmonary bypass or in the setting of postoperative right heart failure. In the setting of severe airway hemorrhage, central VA-ECMO may be required to help minimize blood flow through the lungs to minimize airway bleeding. Furthermore, a bronchial blocker may be placed to help tamponade off the area responsible for bleeding. Waiting several days on central VA-ECMO with a bronchial blocker may help improve the patient’s outcome, or interventional radiology can also be used to help selectively embolize the pulmonary artery branch responsible for the bleeding. Central VA-ECMO can then be decannulated several days later usually. VV-ECMO, however, may be required for patients with either reperfusion pulmonary edema or shunting secondary to steal syndrome, resulting in severe hypoxemia. Either two single-stage cannulas (right internal jugular vein and femoral vein, for example) or a double-stage cannula (right internal jugular vein) can be used for VV-ECMO support. Reversible isolated scenarios such as airway hemorrhage or reperfusion injury are known to have higher chances of decannulation than cases where patients show persistent PH and right ventricular failure.

## 10. Conclusions

PTE continues to be a mainstay in the treatment algorithm for patients with CTEPH. As reviewed here, contemporary surgical management of these patients is complex and requires multidisciplinary coordination in care extending well beyond the operating room and incorporating cardiology, pulmonology, radiology, cardiothoracic surgery, and cardiac anesthesia. Outcome data on CTEPH continues to highlight the safety and efficacy of PTE, which has repeatedly delivered postoperative improvements in hemodynamic measures, pulmonary symptoms, and overall functional status. Importantly, the data presented underscores the association between experience and outcomes in PTE for CTEPH, showcasing the safety of the operation when performed in specialized centers. In experienced hands, PTE remains a powerful and capable operation that can demonstrate profound improvement in the aberrant hemodynamics that are at the core of CTEPH and its associated pathophysiology before even leaving the operating room.

## Figures and Tables

**Table 1:** Comparison of low- vs. high-risk candidates for surgical management of CTEPH. (DVT = deep vein thrombosis; PE = pulmonary embolism; RHF = right heart failure; NYHA = New York Heart Association; PVR = pulmonary vascular resistance; PA = pulmonary artery). Adapted from Ref. [37]. 2019, European Respiratory Society.

Risk	Clinical History	Comorbidities	Imaging Features	Hemodynamics
Lower Risk	+DVT/PE −RHF	None or Minimal NYHA Functional Class II or III	Clear disease, concordance on all studies Bilateral lower lobe distribution	PVR < 1000 dyn·s·cm^−5^ PVR is proportionate to distribution of obstruction on imaging Higher PA pulse pressure
Higher Risk	−DVT/PE +RHF	History of lung and/or left heart disease NYHA Functional Class IV	Inconsistent on imaging studies No lower lobe disease	PVR > 1200 dyn·s·cm^−5^ PVR out of proportion to obstruction on imaging Higher PA diastolic pressure

## Data Availability

No new data were created or analyzed in this study. Data sharing is not applicable to this article.

## Abbreviations

The following abbreviations are used in this manuscript:

CTEPH	chronic thromboembolic pulmonary thromboembolism
PH	pulmonary hypertension
PE	pulmonary embolism
PTE	pulmonary thromboendarterectomy
CTED	chronic thromboembolic disease
RHC	right heart catheterization
CTPA	CT pulmonary arteriography
mPAP	mean pulmonary artery pressure
PVR	pulmonary vascular resistance
DSPA	digital subtraction pulmonary arteriography
CPET	cardiopulmonary exercise testing
MRI	magnetic resonance imaging
BPA	balloon pulmonary angioplasty
DOAC	direct oral anticoagulant
NIRS	near-infrared spectroscopy
BIS	bispectral index
CPB	cardiopulmonary bypass
SVC	superior vena cava
IVC	inferior vena cava
RPA	right pulmonary artery
ECMO	extracorporeal membrane oxygenation
VA-ECMO	veno-arterial extracorporeal membrane oxygenation
VV-ECMO	veno-venous extracorporeal membrane oxygenation
